# Radioembolization for Hepatocellular Carcinoma: a Comparison on Dual-phase Cone-beam CT, Contrast-enhanced CT (CECT) and ^99m^Tc-macroaggregated albumin-SPECT/CT in predicting final distribution volumes and dosimetry of the post-embolization ^90^Y PET/CT

**DOI:** 10.1007/s11547-024-01946-0

**Published:** 2024-12-20

**Authors:** Ettore di Gaeta, Michela Olivieri, Annarita Savi, Patrizia Magnani, Carla Canevari, Simone Gusmini, Diego Palumbo, Giorgia Guazzarotti, Luigi Augello, Francesca Calabrese, Stephanie Steidler, Federica Cipriani, Margherita Rimini, Andrea Casadei-Gardini, Luca Aldrighetti, Arturo Chiti, Francesco De Cobelli

**Affiliations:** 1 Department of Radiology, IRCCS Ospedale San Raffaele, Milan, Italy; 2 Department of Nuclear Medicine, IRCCS Ospedale San Raffaele, Milan, Italy; 3 School of Medicine, Vita-Salute San Raffaele University, Milan, Italy; 4 Department of Hepatobiliary Surgery, IRCCS Ospedale San Raffaele, Milan, Italy; 5 Department of Oncology, IRCCS Ospedale San Raffaele, Milan, Italy

**Keywords:** Transarterial embolization (TARE), Cone-beam computed tomography (CBCT), Hepatocellular carcinoma (HCC), Personalized dosimetry

## Abstract

**Purpose:**

Personalized treatment schemes are being systematically applied to ensure best treatment outcome in oncologic patients. This is true also for personalized dosimetry in transarterial radioembolization (TARE) in hepatocellular carcinoma (HCC) patients. Precise and detailed volumetric and functional data derived from radiological and nuclear imaging methods are essential for personalized dosimetry. We sought to evaluate accuracy of dual-phase cone-beam CT (CBCT) in comparison to pre-treatment contrast-enhanced CT (CECT), and ^99m^Tc-macroaggregated albumin-SPECT/CT ([^99m^Tc]MAA SPECT/CT) to predict and assess the efficacy of TARE based on post-treatment ^90^Y PET/CT.

**Material and methods:**

Thirty consecutive patients with HCC treated with TARE were included. Intraprocedural dual-phase CBCT acquisition protocol was developed to distinguish tumor volume in the early arterial phase and perfused volume of non-affected liver in the late arterial phase. Volumetric data obtained from pre-treatment CECT, dual-phase CBCT and [^99^^m^Tc]MAA SPECT/CT were compared to post-treatment ^90^Y PET/CT considered the standard reference. Treatment simulations for final calculated dose from the different imaging derived volumes were then compared to post-treatment ^90^Y PET/CT.

**Results:**

CBCT resulted as the most accurate method in predicting tumor- (R^2^ 0.89) and perfused volumes (R^2^ 0.84). Dosimetry prediction planning performed on derived volumes from the different methods did not show significant difference, yet highest concordance with ^90^Y PET/CT data was observed with dual-phase CBCT.

**Conclusion:**

Our study shows that dual-phase CBCT acquisition is a novel alternative method for correctly and safely administering more accurate and defined doses during TARE.

*clinicaltrials*.gov ID: NCT03981497.

## Introduction

Effective treatment of hepatocellular carcinoma (HCC) may be challenging and early detection and intervention, along with risk factor management, play a crucial role in improving prognosis for individuals at risk of HCC [1].

Transarterial radioembolization (TARE) with yttrium-90 (^90^Y) has been used as a transition to transplant and palliative care in patients with HCC and recently introduced as definitive treatment for very early stage and early-stage HCC as per BCLC recommendations [2].

A recent randomized clinical trial comparing drug eluting bead transarterial chemoembolization to TARE showed that the latter had a significant reduction in time-to-progression and a better overall survival (respectively, 17.1 months *versus* 9.5 months (0.36 hazard ratio, *p* = 0.002) and 30.2 versus 15.6 months (0.48 hazard ratio, *p* = 0.006)) [3]. These results supported the previously published data comparing the efficacy of TARE, in terms of overall response rate (ORR) and time to progression, to that of chemoembolization in treating single HCC nodules < 8 cm [4].

TARE induced complete or at least partial necrosis of treated lesions that varied significantly based on administered dose [5]. Personalized dosimetry was an important factor in positive outcome also in advanced local disease, increasing median overall survival (OS) from 10.7 to 26.6 months and objective response rate (evaluated 3 months after treatment) from 36 to 76% in subjects in which standard dosimetry was applied [6].

Efficacy of the procedure, therefore, depends on a correct dosimetry planning with accurate tumor volume (TV) and perfusion volume (PV) segmentation, yet debate remains on how different imaging techniques and artifacts may influence these evaluations. [^99m^Tc]MAA-SPECT/CT has been demonstrated to be more accurate than single-phase cone-beam computed tomography (CBCT) in predicting final tumor volume [6, 7], but CBCT is essential in correct dosimetry and in patients with multiple feeders [6]. The European Association of Nuclear Medicine (EANM) 2021 and 2022 guidelines detail the use of contrast-enhanced CT (CECT) for tumor volume segmentation and SPECT/CT for evaluating the perfused region [8, 9] as best suggested approach.

In standard practice [10–12], CBCT consists in a single early arterial phase for assessment of lesion feeding arteries to guide super selective catheterization, confirm tumor load and treatment indication. Dual-phase CBCT acquires images during the early and late arterial phases depicting tumor volume and perfused parenchyma [13] with superior accuracy in tumor detection versus single-phase imaging [13–15].

The aim of this study was to evaluate if pre-treatment contrast-enhanced CT, dual-phase cone-beam CT (early and late arterial phase) and [^99m^Tc]-macroaggregated albumin-SPECT/CT accurately predict volume and ^90^Y-resin-microsphere activity distribution compared to post-embolization reference standard ^90^Y PET/CT.

## Material and methods

**Fig. 1:**
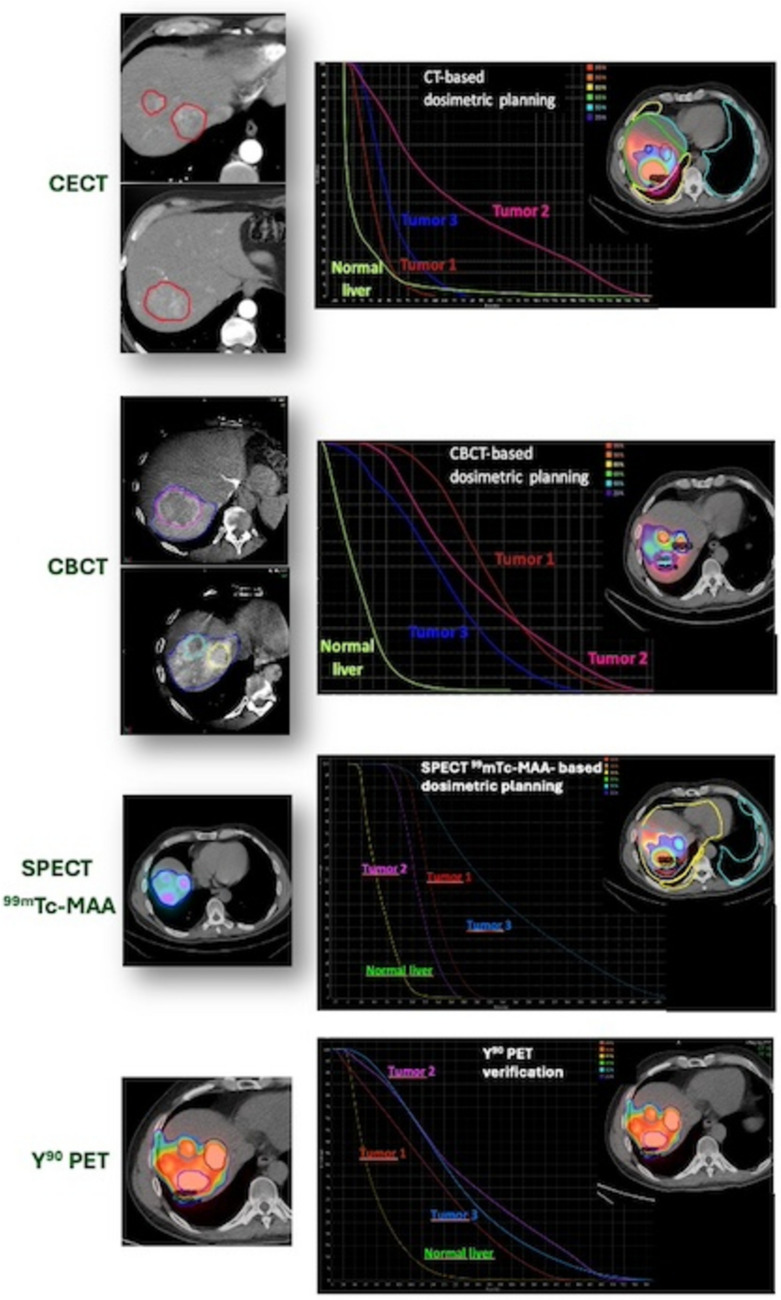
*Consecutio* of the different study phases: tumor and perfused volume segmentation, the derived volumes co-registered with [^99m^Tc]MAA SPECT for treatment simulation and verification with ^90^YPET

Following patient selection, tumor and perfused volumes were segmented on baseline contrast-enhanced CT and dual-phase CBCT and co-registered to [^99m^Tc]-MAA SPECT/CT. The values obtained on the different imaging modalities were used for treatment simulation and then were compared to those obtained on reference standard post-embolization ^90^Y PET/CT. Details are further described in each section (Fig. 1).

### Patient population

Thirty-six lesions of 30 HCC patients that underwent TARE with resin ^90^Y-microspheres (SIR-spheres® SIRTex Medical Limited) from January 2020 to August 2023 were collected prospectively and retrospectively evaluated. Pre-procedural contrast-enhanced CT (CECT) performed within 30 days from procedure was primary inclusion criteria. Therapeutic indication for TARE was discussed and agreed in the dedicated multidisciplinary team prior to treatment. Patient data was collected after having signed a specific informed consent previously approved by the Institutions’ Ethical Committee (64/INT/2021).

### Radioembolization protocol

Workup and treatment were performed following the current standard of practice at our Institution.

Radioembolization was divided into two sessions performed approximately one week apart. During the first session, mapping angiography and intraprocedural dual-phase CBCT was performed. The acquisition protocol was divided in an early and late arterial phase, to identify, respectively, the target lesion, its feeders and surrounding perfused parenchyma. Upon correct arterial access selection, [^99m^Tc]macroaggregated albumin was injected to evaluate lung and gastrointestinal shunt fraction and Tumor to Normal tissue uptake ratio (TNR) [13]. ^90^Y activity calculation was based on a multicompartmental model [16], considering a tumor absorbed dose > 120 Gy, overall normal liver dose < 40 Gy and a lung dose < 30 Gy [9] [13] [16]. Prior to administration of ^90^Y-spheres, patients underwent a confirmatory angiography and CBCT to evaluate any change in perfusion. Standard of care confirmatory ^90^Y PET/CT study is acquired within 24 h after treatment.

### Imaging acquisition protocols

#### Contrast-Enhanced CT

Upper and lower abdominal scans were acquired on multidetector CT scanners (Siemens Somatom definition flash Syngo or Philips Brilliance 64). The scan included a non-contrast and a triple-phase acquisition to evaluate focal liver lesions (late arterial, portal and delayed). The arterial phase (30 s) was used to evaluate viable tumor tissue and vessels anatomy and to calculate tumor volume (TV).

#### Dual-Phase Cone-Beam CT Technique

Acquisition of images was performed using an angiographic system (Azurion 7 C20, Philips Healthcare) equipped with the XperCT Dual option, that allows dual-phase rotational acquisition (early and late arterial phases) with a single contrast injection. The C-arm rotation in head-end position (120°LAO- 185°RAO) reaches 25°/sec at a voltage of 120 kVp with a detector size of 12 inches; total rotation time is 5.2 s.

**Fig. 2:**
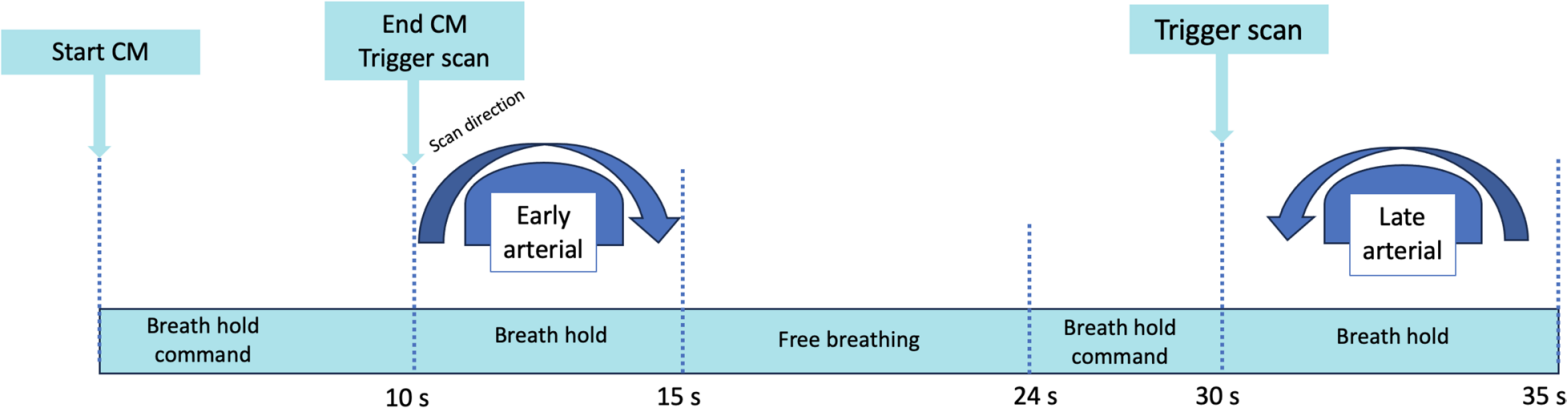
Dual-Phase CBCT acquisition protocol

**Fig. 3:**
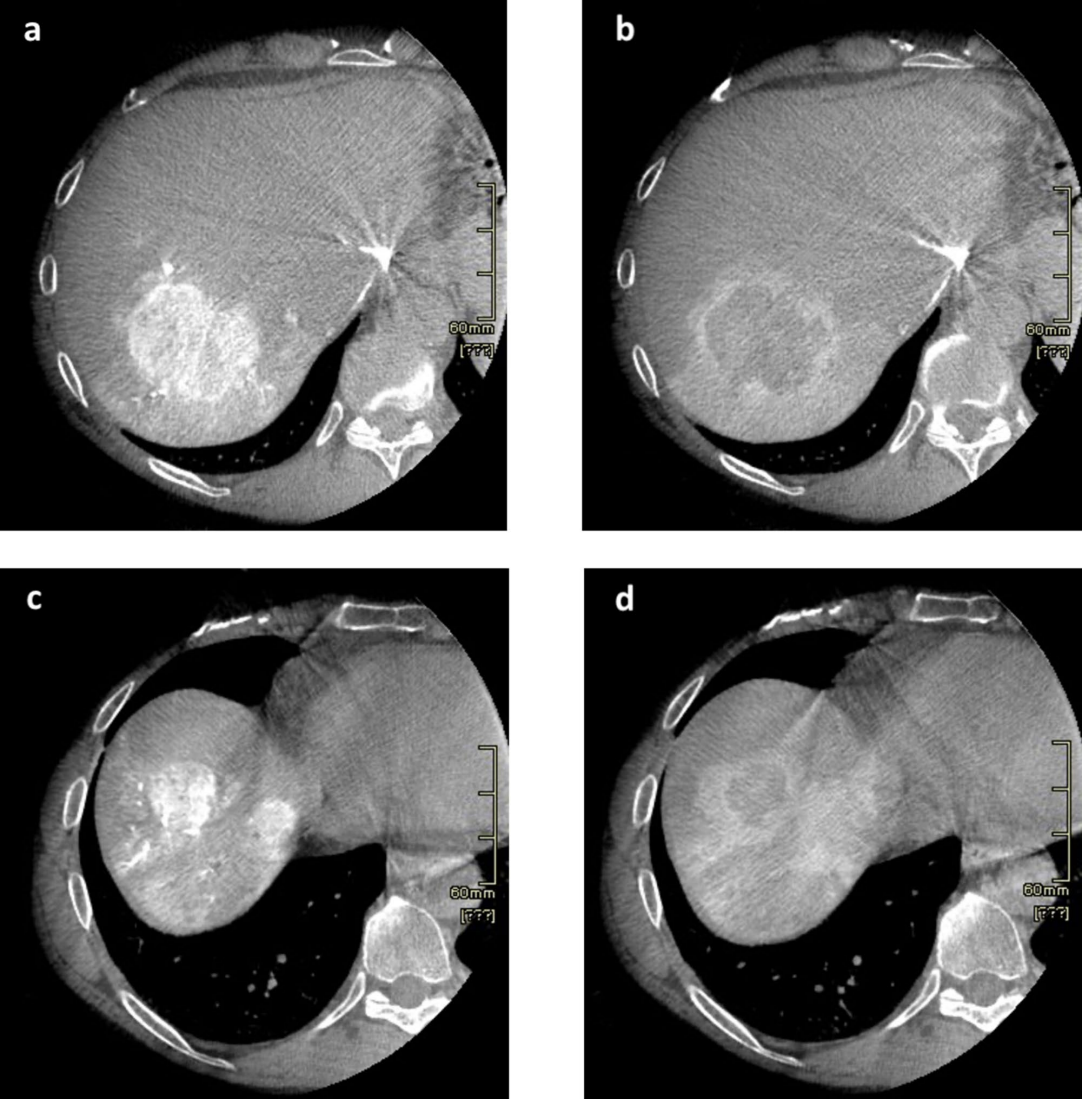
Dual-phase CBCT: **a** Early arterial phase (10–15 s) Lesion 1; **b** Late arterial phase (30 s) Lesion 1; **c** Early arterial phase (10–15 s) Lesion 2 and 3; **d** Late arterial phase (30 s) Lesion 2 and 3

A specifically developed contrast agent injection protocol (Ultravist, 370 mg of iodine per milliliter) was standardized to deliver 10 mL at a rate of 1 mL/second in segmental branches of the hepatic artery and acquisition was performed during end expiration apnea. In cases of superselective catheterism of subsegmental branches, the injection protocol ratio remained unvaried, at a rate of 0,5 mL/second for 5 mL administered. The early arterial phase was triggered at 10 s after contrast injection and used to evaluate tumor volume; the late arterial phase, at 15–20 s after the first acquisition, for perfusion volume (Fig. 2). This injection protocol allows evaluation of both the feeding arteries and the hyper-vascular lesion/s as contrast lasts throughout the first acquisition. The perfused parenchyma is then visible in the late arterial phase (approximately at a delay of 30 s) (Fig. 3). The low rate of injection reduces the risk of contrast reflux to non-target areas. Automated image reconstruction is visible after completion of each scan.

##### ^*[99m*^*Tc]macroaggregated albumin-SPECT/CT ([*^*99m*^*Tc]-MAA SPECT/CT*

Whole body planar scintigraphy (from thyroid to bladder) was acquired in anterior and posterior projections followed by a SPECT/CT scan (128 × 128, 120 steps, 20 s/step Discovery NM 670, GE Healthcare) within 30 min after conclusion of the angiography procedure. Planar images were used to define lung shunt fraction and presence of gastrointestinal shunt. SPECT data was obtained with a 3D ordered-subset expectation maximization (4 iterations, 10 subsets) with a Butterworth filter (cutoff,0.5 cycles/cm; order, 10) and CT-based attenuation, scatter correction and resolution recovery.

##### ^*90*^*Y PET/CT*

Two bed positions of 15 minutes each, including complete liver coverage, were acquired on a Time Of-Flight (TOF) PET/CT system (Discovery PET/CT 690, GE Healthcare). PET data was reconstructed using a Bayesian reconstruction algorithm (VPFX, 2iterations 16 subsets) with a gaussian post-reconstruction filter of 5 mm in full width at half maximum including time of flight information. Normalization, dead time, activity decay, random coincidences, attenuation, scatter and resolution recovery (imaging corrections) were used.

### Tumor and perfused volume delineation

All tumor- and perfused volumes (TV, PV) and overall healthy total liver volume were segmented using MIM software (Beachwood, Ohio- 7.2.8). Segmentation was performed using a semi-automated method which was then corrected manually, if needed.

TV on CECT and PV and TV on CBCT were evaluated by two interventional radiologists (EDG and LA). TV and PV from [^99m^Tc]MAA SPECT/CT and ^90^Y PET/CT were evaluated by a nuclear medicine physician, using a threshold method.

Tumor volumes, defined as the viable tumoral tissue showing uptake in arterial phase, were retrospectively segmented on the arterial phase of CECT and early phase of intraprocedural CBCT. Necrotic areas were excluded from volume delineation.

TVs were also delineated on [^99m^Tc]MAA SPECT/CT and ^90^Y PET/CT images using a threshold method. Delineation was carried out on the fusion images (SPECT and CT, PET and CT) and for each volume, the threshold value was visually adjusted in order to match the contours based on the activity distribution with the CT lesion borders. Mean threshold value for TVs on [^99m^Tc]MAA SPECT/CT and ^90^Y-PET/CT images was 30% [16].

Perfusion volumes (defined as the parenchyma of the segment vascularized by the arterial branch selectively catheterized) including the lesion and the surrounding healthy parenchyma enhanced following contrast injection, were segmented on one or more (in case of multiple feeding arteries) late arterial phases of intraprocedural CBCT acquisition.

Due to the broad distribution of single phase CBCT protocols, PV was also segmented in the early phase to confirm accuracy compared to the late arterial phase. In addition, PVs were delineated on [^99m^Tc]MAA SPECT/CT and ^90^Y PET/CT images using a threshold method. The mean threshold value for PVs on [^99m^Tc]MAA SPECT/CT and ^90^Y PET/CT images was 5% [7].

### Treatment planning simulation and verification

TVs and PVs delineated on CECT and CBCT were transferred to the SPCET/CT images using non-rigid co-registration. Treatment planning simulations were performed independently considering volumes segmented on the three imaging modalities: CECT, CBCT and [^99m^Tc]MAA SPECT/CT. For CECT alone, the overall healthy total liver was used to calculate perfused volume toxicity.

Fixed tumor- and perfused parenchyma doses were set at 120 Gy and 40 Gy, respectively. Simulations of PET dose distribution were evaluated by normalizing PET images to the three fictitious activities derived from the treatment planning obtained with volumes delineated on the three imaging modalities. All phases of the treatment planning simulation were performed using the MIM software.

### Comparisons

^90^Y PET/CT volumes were considered as reference standards. TVs obtained on CECT, CBCT early arterial phases and [^99m^Tc]MAA SPECT/CT were compared to those obtained on post-embolization ^90^Y PET/CT. PVs from CBCT early and late arterial phases and [^99m^Tc]MAA SPECT/CT were compared to those obtained on post-embolization ^90^Y PET/CT. Early arterial dual-phase CBCT, CECT and [^99^^m^Tc]MAA-SPECT/CT were compared head-to-head using a one-way ANOVA. In order to evaluate the agreement of the predicted dosimetry derived from the treatment planning simulations and post-treatment dosimetry, the dosimetric metrics considered were V120Gy, defined as the percentage of target volume receiving at least 120 Gy, and V40Gy, percentage of healthy parenchyma volume receiving 40 Gy [9].

### Statistical analysis

Statistical analysis was performed using MedCalc software (Medcalc Software Ltd, Ostend, Belgium – v20.216). The coefficient of determination R^2^ of the regression model was calculated to compare PVs and TVs derived in CECT, CBCT and [^99m^Tc]MAA SPECT/ to those obtained on post-embolization ^90^YPET/CT. Interobserver agreement (for tumor and perfused volume) on all imaging was evaluated using the Dice coefficient. Correlation of volumes obtained between techniques is depicted using Bland–Altman plots. Quantitative descriptive statistics are expressed as medians and interquartile ranges; tumor size is reported in mean and standard deviation. A p-value less than 0.05 was considered significant. Head-to-head comparisons among modalities was evaluated with one-way ANOVA. Agreement between V120Gy and V40Gy obtained in CECT, CBCT, SPECT, values were calculated on PET normalized imaging. Concordance correlation coefficient (CCC) for dose comparison were calculated with a 95% Confidence Interval (CI).

## Results

**Fig. 4:**
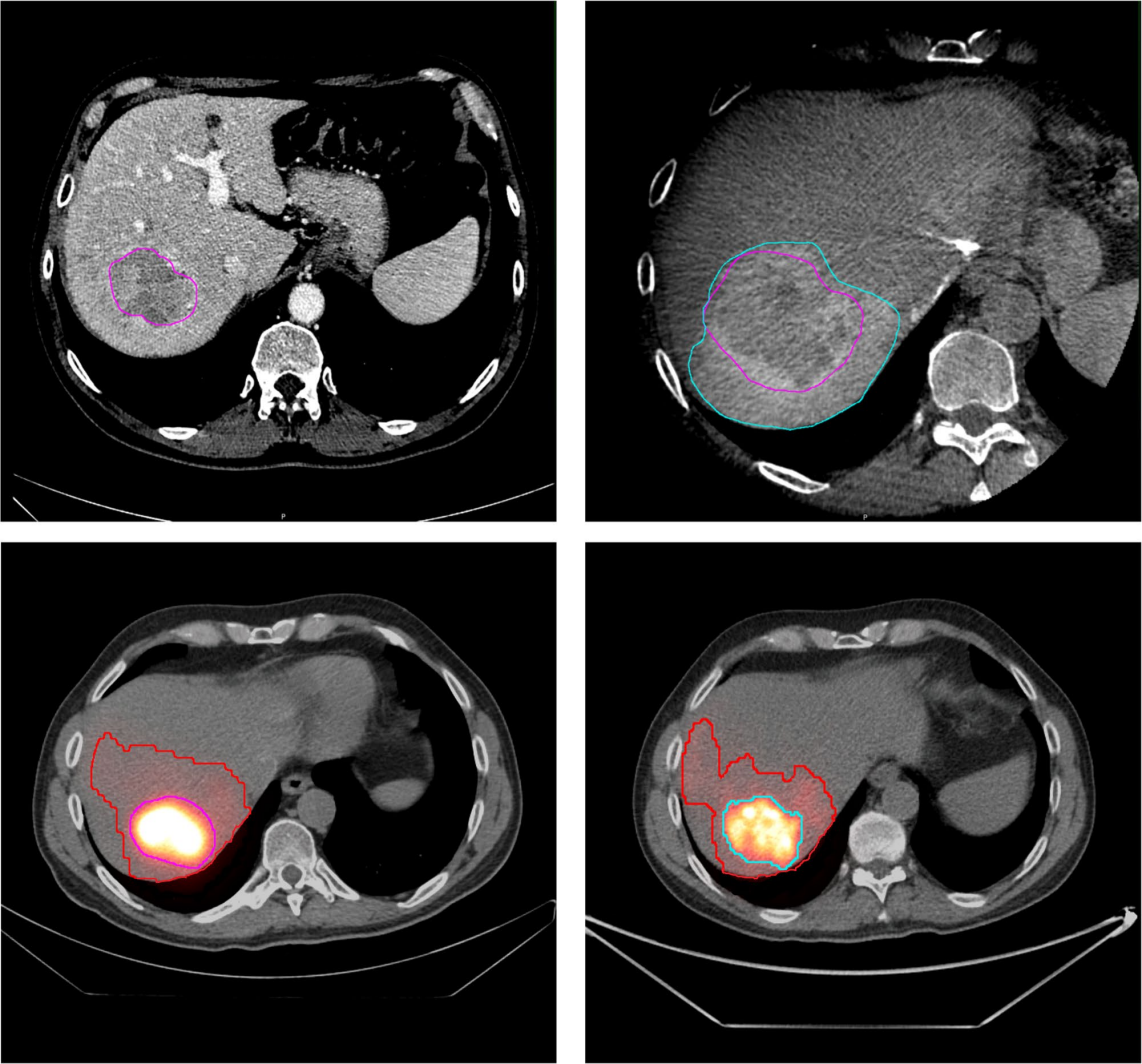
TV and PV segmentation in different imaging modalities a) CECT portal phase, b) CBCT late arterial phase c) 99mTc-MAA-SPECT, d) 90Y PET/CT

Most patients were males (*n* = 28) with Child B stage liver predominant disease (*n* = 17). Seven patients had multiple tumors treated in the same session. Patient data is described in Table 1. No major peri-procedural or post-procedural (up to discharge, 2 days post procedure) complications were observed. Interobserver Dice coefficient among the two readers was 0.88 for tumor volume and 0.85 for perfused volume, therefore subsequent calculations were derived from data of one single reader. An example of perfusion- and tumor volume segmentation on the four different imaging modalities are depicted in Fig. 4.

**Table 1:** Population demographic data

Number of patients	30
Age at TARE (mean ± SD)	74 (14.7)
*Gender, n (%)*	
Male	28 (93.33%)
Female	2 (6.66%)
*BCLC*	
BCLC A	9 (30%)
BCLC B	17 (56.7%)
BCLC C	4 (13.3%)
*Child- Pugh Score*	
A	26 (86.7%)
B	4 (13.3%)
Baseline tumor size (mean ± SD)	52.5 ± 24.72 mm

HCC, hepatocellular carcinoma; BCLC, Barcelona Clinic Liver Cancer; SD, standard deviation

**Fig. 5:**
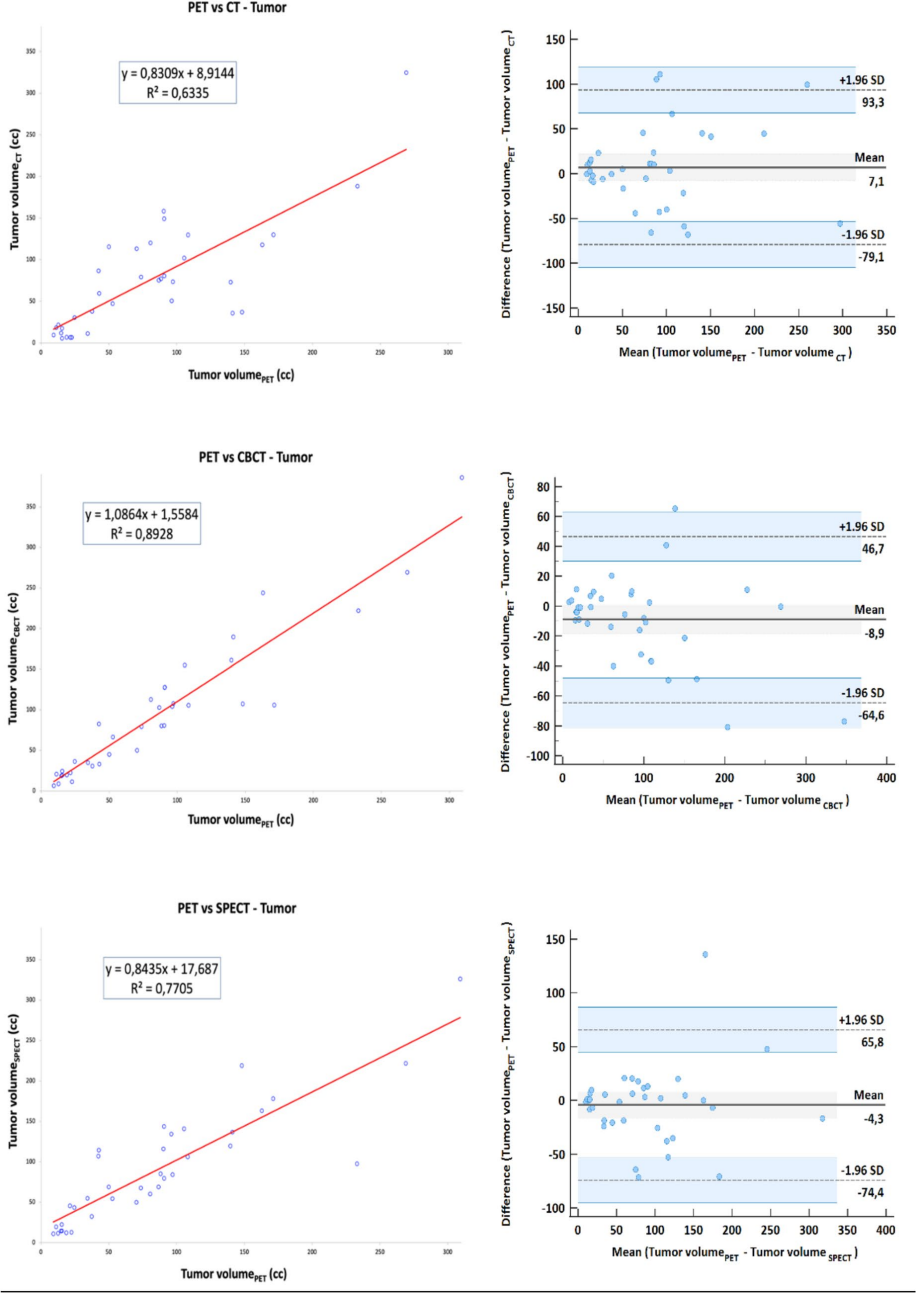
Correlation (right) and agreement (left) derived from TVs delineated on **a** CECT, **b** early arterial phase CBCT, **c** 99mTc-MAA SPECT/CT and PET

Median TVs and PVs are described in Table 2. No statistically significant differences between volumes calculated per different imaging modalities were obtained. No significant differences were found in a direct head-to-head comparison comparing CECT to dual-phase CBCT early arterial phase and [^99^mTc] MAA-SPECT/CT for predicting tumor volume (one-way ANOVA followed by Tukey’s test CBCT vs SPECT p = 0.8034; CBCT vs CECT p = 0.2052; SPECT vs CECT p = 0.4366). TV correlation between modalities and agreement (according to Bland–Altman) is depicted in Fig. 5.

**Table 2:** Tumor (TV) and perfusion volumes (PV) per different imaging modalities

	TV (cc) median (range)	PV (cc) median (range)
CECT	77.84 (6.89–324.92)	n.c
CBCT	95.6 (6.48–386.17)	445.10 (19.19–960) early arterial phase
		321.03 (14.74–960) late arterial phase
SPECT	89.39 (10.77–326.03)	466.10 (24–1171.55)
^90^Y PET	92.63 (9.18–309.17)	427.48 (38.76–961.22)
n.c.- not calculated

**Fig. 6:**
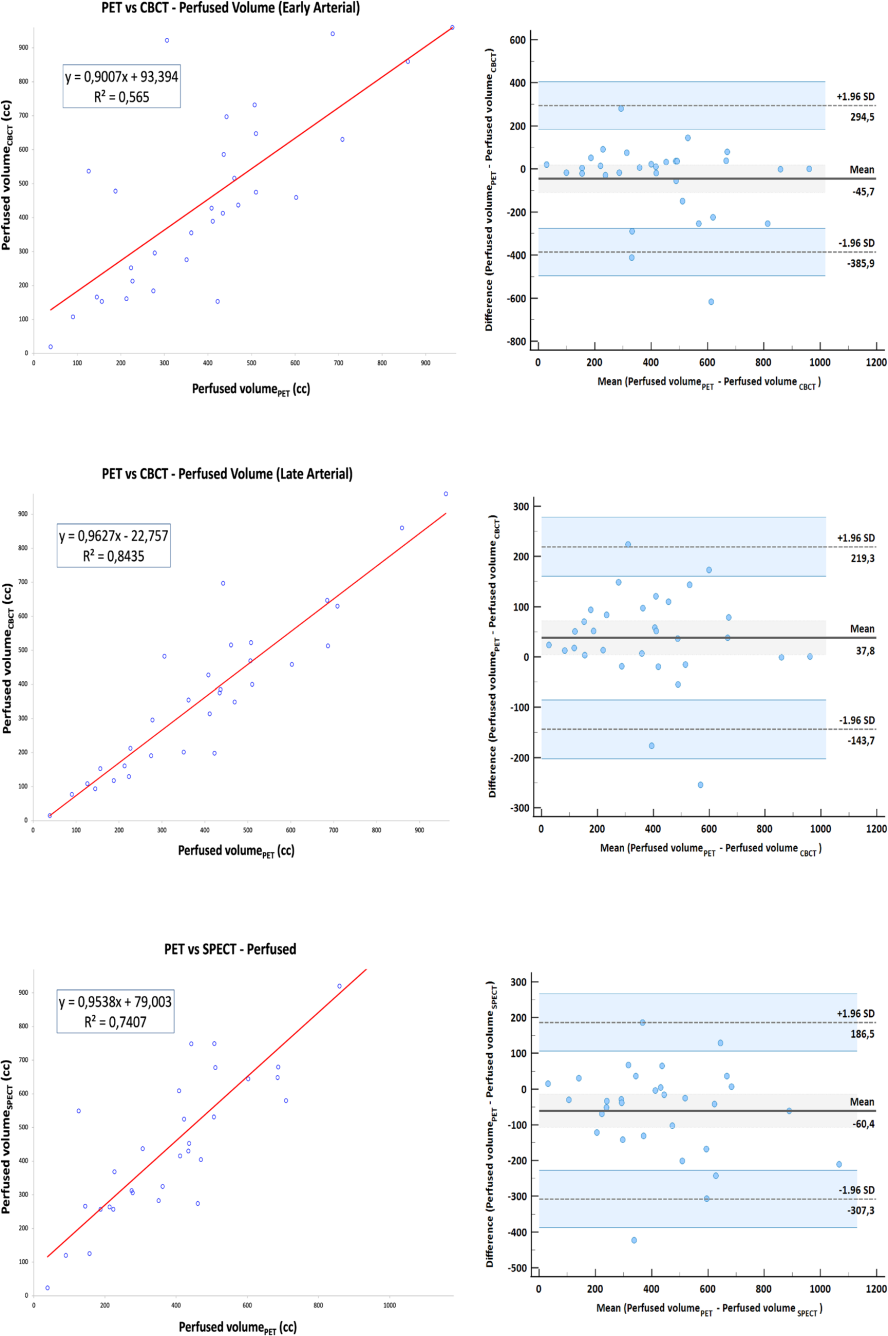
Correlation (right) and agreement (left) derived from PVs delineated on **a** early arterial phase CBCT, **b** late arterial phase CBCT, **c** [^99m^Tc]MAA SPECT/CT and PET-based volumes

PV correlation in early arterial phase CBCT, late arterial phase CBCT, [^99m^Tc] MAA SPECT/CT and PET-based volumes and agreement are depicted in Fig. 6.

Early arterial CBCT and late arterial CBCT provided the best R^2^ (0.89, 0.84) for TVs and PVs, respectively. [^99m^Tc]MAA SPECT/CT showed a better correlation with PET for tumor volume (R^2^ = 0.77) versus CECT. Simulation of median activities based on a tumor dose of 120 Gy derived from TV/PV on the different imaging modalities are described in Table 3. No statistically significant difference between the calculated activities was observed.

**Table 3:** The percent volume at 120 Gy and 40 Gy and concordance correlation coefficient with PET of the different imaging techniques

Imaging technique	Activity (GBq)	V_120Gy_ (%)	V_40Gy_ (%)	CCC (V_120Gy_)	CCC (V_40Gy_)
CECT	0.70 (0.30–1.10)	36.11 (7.90–56.39)	20.9 (3.2–40.7)	0.64 (0.14–0.88)	0.24 (0.07–0.52)
CBCT	0.70 (0.20–1.00)	23.24 (5.30–54.46)	23.51 (1.00–67.87)	0.85 (0.57–0.96)	0.70 (0.06–0.93)
[^99m^Tc]MAA SPECT/CT	0.50 (0.20–1.10)	21.42 (10.70–40.86)	19.39 (4.09–41.95)	0.75 (0.35–0.92)	0.49 (0.18–0.85)

Best agreement was found both for V120Gy and V40Gy calculated on CBCT and SPECT versus CECT.

## Discussion

The benefit of personalized dosimetry in TARE has been demonstrated by several authors [7, 17–19]. Accurate calculation of target and perfused volumes is mandatory due to the direct impact on absorbed dose estimation [20]. To the best of our knowledge, this is the first study that evaluates tumor- and perfused volumes with dual-phase cone-beam CT in comparison to contrast-enhanced CT (CECT) and [^99m^Tc] macroaggregated albumin-SPECT/CT ([^99m^Tc]MAA SPECT/CT) versus ^90^Y distribution on standard of reference PET/CT. ^90^Y PET is a well-established technique for ^90^Y treatment verification after selective internal radiation therapy (SIRT) as it provides improved accuracy for dosimetry [21] and reflects tumor heterogeneity [22] Our dual-phase cone-beam computed tomography (CBCT) acquisition protocol warrants more precise volumetric assessment and more accurate definition of tumor and perfused volume. This may be of particular interest in patients with multiple lesions and/or multiple feeders (super selective catheterization) or in patients with infiltrative diseases.

Our results demonstrate that late arterial phase cone beam computed tomography (CBCT), obtained by our standardized and dedicated injection protocol, more accurately predicts perfusion volume, as defined in ^90^Y PET/CT, than early arterial phase CBCT and [^99m^Tc]MAA-SPECT/CT. This result must not be considered divergent from what previously reported by Rodríguez‐Fraile et al. [7], in which that [^99m^Tc]MAA-SPECT/CT was more accurate than single-phase CBCT, since single arterial phase acquisition used for perfusion volume delineation can produce errors in volume estimation due to reflux artifacts [7].

Contrast-enhanced CT and SPECT have been extensively used for tumor volume assessment [9, 23]. The debate about the correlation between activity distribution of [^99m^Tc]MAA and ^90^Y microspheres is still open. Discrepancies are related to the number, density, size, and morphology differences between MAA and spheres or the ability to administer both compounds under identical conditions [24, 25]. Despite these limitations several authors have demonstrated a good correlation between [^99m^Tc]MAA and 90Y microspheres distribution [26, 27]. Recently, alternatives have been proposed to overcome these above-mentioned limitations such as low dose ^90^Y [28] or Ho-166 [29] as scout dose nevertheless, [^99m^Tc] MAA SPECT is routinely used in the clinical setting and is readily available.

Our results showed that early arterial phase CBCT yields a more precise volumetric assessment most likely due to the higher spatial resolution (versus [^99m^Tc]SPECT/CT) and ability to distinguish and exclude necrotic areas. Median tumor volumes on CECT were lower than those obtained on CBCT and other imaging modalities contrary to what described by other authors [9, 23], most likely due to the different timing of the acquisition. Stein et al. [30] found that CBCT yielded a more accurate tumor volume dose estimation in the diagnostic phase than pre-procedural CT and MRI in patients planned for segmentectomy. High resolution planning CBCT has also been applied in a computational tool used to segment the arterial tree and predict distribution [31]. Leveraging the strengths of CBCT in segmentation and tumor volume evaluation may therefore aid in more precise dosimetry. The best agreement between tumor and non-tumoral liver ratio, V120Gy and V40Gy, respectively, threshold for complete response and healthy liver, and post-treatment dosimetry was also derived from CBCT; even if not statistically significant, it outperformed other modalities resulting in an additional asset for its use in personalized treatment approaches.

The following limitations need to be considered. Data collected prospectively from a single institution and retrospectively evaluated resulted in a limited number of patients for this cohort. Cone beam CT may include smaller fields of view with limited liver volume recognition or artifacts caused by breath hold in which acquisitions need to be repeated. Errors in co-registration of imaging techniques may also play a role in incorrect delineation. CBCT cannot identify extrahepatic activity and lung shunts and will therefore need to be integrated with other imaging modalities. Reference standard ^90^Y PET/CT calculations may be biased by noisy imaging and the use of threshold methods, even though still used as reference standards, may underestimate necrotic areas [22, 26].

## Conclusion

Dual-phase cone beam computed tomography (CBCT) predicts both TVs and PVs more accurately and yields a better concordance correlation coefficient with ^90^Y PET/CT; [^99m^Tc]MAA-SPECT showed a good agreement with ^90^Y PET/CT in terms of volume and remains essential to correctly derive the Tumor/Nontumor uptake ratio; SPECT also remains necessary for identifying potential pulmonary shunts and possible activity outside the liver (extrahepatic activity). CBCT becomes fundamental in case of multiple feeding arteries in which a more accurate dose split is necessary. Volumes derived from CBCT give the best prediction of actual distribution allowing a more accurate and personalized dosimetry planning in TARE.
