# Correction: Radioembolization for Hepatocellular Carcinoma: a Comparison on Dual-phase Cone-beam CT, Contrast-enhanced CT (CECT) and ^99m^Tc-macroaggregated albumin-SPECT/CT in predicting final distribution volumes and dosimetry of the post-embolization ^90^Y PET/CT

**DOI:** 10.1007/s11547-025-02031-w

**Published:** 2025-06-23

**Authors:** Ettore di Gaeta, Michela Olivieri, Annarita Savi, Patrizia Magnani, Carla Canevari, Simone Gusmini, Diego Palumbo, Giorgia Guazzarotti, Luigi Augello, Francesca Calabrese, Stephanie Steidler, Federica Cipriani, Margherita Rimini, Andrea Casadei-Gardini, Luca Aldrighetti, Arturo Chiti, Francesco De Cobelli

**Affiliations:** 1 Department of Radiology, IRCCS Ospedale San Raffaele, Milan, Italy; 2 Department of Nuclear Medicine, IRCCS Ospedale San Raffaele, Milan, Italy; 3 School of Medicine, Vita-Salute San Raffaele University, Milan, Italy; 4 Department of Hepatobiliary Surgery, IRCCS Ospedale San Raffaele, Milan, Italy; 5 Department of Oncology, IRCCS Ospedale San Raffaele, Milan, Italy

Correction to: La radiologia medica (2024) 130:474–485 10.1007/s11547-024-01946-0

In the published version, author has found errors in the Abstract section:

Published version: Purpose Personalized treatment schemes are being systematically applied to ensure best treatment outcome in oncologic.

patients. This is true also for personalized dosimetry in transarterial radioembolization (TARE) in hepatocellular carcinoma.

(HCC) patients. Precise and detailed volumetric and functional data derived from radiological and nuclear imaging methods.

are essential for personalized dosimetry. We sought to evaluate accuracy of dual-phase cone-beam CT (CBCT) in comparison.

to pre-treatment contrast-enhanced CT (CECT), and 99mTc-macroaggregated albumin-SPECT/CT ([

99mTc]MAA SPECT/

CT) to predict and assess the efficacy of TARE based on post-treatment 90Y PET/CT.

Material and methods Thirty consecutive patients with HCC treated with TARE were included. Intraprocedural dual-phase.

CBCT acquisition protocol was developed to distinguish tumor volume in the early arterial phase and perfused volume of nonaffected.

liver in the late arterial phase. Volumetric data obtained from pre-treatment CECT, dual-phase CBCT and [99mTc].

MAA SPECT/CT were compared to post-treatment 90Y PET/CT considered the standard reference. Treatment simulations.

for final calculated dose from the different imaging derived volumes were then compared to post-treatment 90Y PET/CT.

Results CBCT resulted as the most accurate method in predicting tumor- (

R2 0.88) and perfused volumes (

R2 0.82). Dosimetry.

prediction planning performed on derived volumes from the different methods did not show significant difference.

(p < 0.05), yet highest concordance with 90Y PET/CT data was observed with dual-phase CBCT.

Conclusion Our study shows that dual-phase CBCT acquisition is a novel alternative method for correctly and safely administering.

more accurate and defined doses during TARE.

clinicaltrials.gov ID: NCT03981497.

Correct version: Purpose Personalized treatment schemes are being systematically applied to ensure best treatment outcome in oncologic.

patients. This is true also for personalized dosimetry in transarterial radioembolization (TARE) in hepatocellular carcinoma.

(HCC) patients. Precise and detailed volumetric and functional data derived from radiological and nuclear imaging methods.

are essential for personalized dosimetry. We sought to evaluate accuracy of dual-phase cone-beam CT (CBCT) in comparison.

to pre-treatment contrast-enhanced CT (CECT), and 99mTc-macroaggregated albumin-SPECT/CT ([

99mTc]MAA SPECT/

CT) to predict and assess the efficacy of TARE based on post-treatment 90Y PET/CT.

Material and methods Thirty consecutive patients with HCC treated with TARE were included. Intraprocedural dual-phase.

CBCT acquisition protocol was developed to distinguish tumor volume in the early arterial phase and perfused volume of nonaffected.

liver in the late arterial phase. Volumetric data obtained from pre-treatment CECT, dual-phase CBCT and [99mTc].

MAA SPECT/CT were compared to post-treatment 90Y PET/CT considered the standard reference. Treatment simulations.

for final calculated dose from the different imaging derived volumes were then compared to post-treatment 90Y PET/CT.

Results CBCT resulted as the most accurate method in predicting tumor- (

R2 0.89) and perfused volumes (

R2 0.84). Dosimetry.

prediction planning performed on derived volumes from the different methods did not show significant difference.

yet highest concordance with 90Y PET/CT data was observed with dual-phase CBCT.

Conclusion Our study shows that dual-phase CBCT acquisition is a novel alternative method for correctly and safely administering.

more accurate and defined doses during TARE.

clinicaltrials.gov ID: NCT03981497.

